# Evaluation of early-phase [^18^F]-florbetaben PET acquisition in clinical routine cases

**DOI:** 10.1016/j.nicl.2016.10.005

**Published:** 2016-10-08

**Authors:** Sonja Daerr, Matthias Brendel, Christian Zach, Erik Mille, Dorothee Schilling, Mathias Johannes Zacherl, Katharina Bürger, Adrian Danek, Oliver Pogarell, Andreas Schildan, Marianne Patt, Henryk Barthel, Osama Sabri, Peter Bartenstein, Axel Rominger

**Affiliations:** a Dept. of Nuclear Medicine, Ludwig-Maximilians-Universität München, München, Germany; b ISD, Ludwig-Maximilians-Universität München, München, Germany; c Dept. of Neurology, Ludwig-Maximilians-Universität München, München, Germany; d Dept. of Psychiatry, Ludwig-Maximilians-Universität München, München, Germany; e Dept. of Nuclear Medicine, University of Leipzig, Leipzig, Germany; f SyNergy, Ludwig-Maximilians-Universität München, München, Germany; g German Center for Neurodegenerative Diseases (DZNE), Munich, Germany

**Keywords:** PET, Positron emission tomography, FBB, [^18^*F*]florbetaben, FDG, [^18^F]-fluorodeoxyglucose, 3D-SSP, 3-dimensional stereotactic surface projections, p.i., post injection, AD, Alzheimer's disease, SPECT, single photon emission computed tomography, CBF, cerebral blood flow, CBL, cerebellum, GLM, global mean, MNI, Montreal Neurological Institute, SUVR, standardized uptake value ratio, VOI, volume of interest, PCC, posterior cingulate cortex, R, right, L, left, FTLD, frontotemporal lobar degeneration, MCI, mild cognitive impairment, CN, cognitively normal, Alzheimer's disease, ß-amyloid, [^18^F]-florbetaben PET, FDG Pet, Metabolism, Perfusion

## Abstract

**Objectives:**

In recent years several [^18^F]-labelled amyloid PET tracers have been developed and have obtained clinical approval. There is accumulating evidence that early (post injection) acquisitions with these tracers are equally informative as conventional blood flow and metabolism studies for diagnosis of Alzheimer's disease, but there have been few side-by-side studies. Therefore, we investigated the performance of early acquisitions of [^18^F]-florbetaben (FBB) PET compared to [^18^F]-fluorodeoxyglucose (FDG) PET in a clinical setting.

**Methods:**

All subjects were recruited with clinical suspicion of dementia due to neurodegenerative disease. FDG PET was undertaken by conventional methods, and amyloid PET was performed with FBB, with early recordings for the initial 10 min (early-phase FBB), and late recordings at 90–110 min p.i. (late-phase FBB). Regional SUVR with cerebellar and global mean normalization were calculated for early-phase FBB and FDG PET. Pearson correlation coefficients between FDG and early-phase FBB were calculated for predefined cortical brain regions. Furthermore, a visual interpretation of disease pattern using 3-dimensional stereotactic surface projections (3D-SSP) was performed, with assessment of intra-reader agreement.

**Results:**

Among a total of 33 patients (mean age 67.5 ± 11.0 years) included in the study, 18 were visually rated amyloid-positive, and 15 amyloid-negative based on late-phase FBB scans. Correlation coefficients for early-phase FBB vs. FDG scans displayed excellent agreement in all target brain regions for global mean normalization. Cerebellar normalization gave strong, but significantly lower correlations. 3D representations of early-phase FBB visually resembled the corresponding FDG PET images, irrespective of the amyloid-status of the late FBB scans.

**Conclusions:**

Early-phase FBB acquisitions correlate on a relative quantitative and visual level with FDG PET scans, irrespective of the amyloid plaque density assessed in late FBB imaging. Thus, early-phase FBB uptake depicts a metabolism-like image, suggesting it as a valid surrogate marker for synaptic dysfunction, which could ultimately circumvent the need for additional FDG PET investigation in diagnosis of dementia.

## Highlights

• Early-phase [^18^F]-florbetaben uptake depicts a metabolism-like image
• Strong relative quantitative and visual correlations of early-phase [^18^F]-florbetaben uptake with FDG images
• A two-phase [^18^F]-florbetaben protocol might give combined neurodegeneration and amyloid pathology biomarker information
• Early-phase [^18^F]-florbetaben PET could ultimately circumvent the need for an additional FDG-PET in the dementia work-up.

## 1 Introduction

As the most prevalent form of neurodegenerative dementias, Alzheimer's disease (AD) is imposing an onerous burden on health care systems in societies with aging populations (Ziegler-Graham et al., 2008). Intracellular neurofibrillary tangles and extracellular amyloid plaques together comprise the hallmark neuropathology of AD (Braak and Braak, 1991). Elevated brain amyloid burden is associated with cognitive decline in cognitively normal (CN) subjects (Lim et al., 2012), and in cases of mild cognitive impairment (MCI), who are at high risk for conversion to AD in a matter of years (Lim et al., 2014). Recently, amyloid PET radiotracers such as [^18^*F*]florbetaben (FBB) have been developed, and have proven to be sensitive indicators for brain amyloid pathology in vivo (Barthel and Sabri, 2011). Amyloid plaques play a role in early pathogenesis of AD, and may even be present 10–15 years prior to onset of discernible cognitive decline, before developing to a stable level observed at the clinical stages of AD (Kadir et al., 2012). Thus, the extensive amyloid accumulation during the pre-clinical course may disfavor the use of FBB and related PET tracers to determine the extent of neurodegeneration or to monitor disease progression in clinical stages of AD (Furst et al., 2012). In contrast, findings with more conventional [^18^*F*]fluorodeoxyglucose (FDG) PET for measuring cerebral glucose metabolism, or perfusion SPECT scans, are a much more sensitive indicator for disease stage, and can provide information about synaptic dysfunction and the degree of neurodegeneration (Herholz, 2011, Shokouhi et al., 2013).

In addition to these considerations, positive amyloid burden is seen not only in AD but also in other neurodegenerative dementias, notably in a subset of patients with dementia with Lewy bodies or Parkinson's disease dementia (Donaghy et al., 2015). Accordingly, additional FDG PET or perfusion SPECT is considered beneficial for differentiating amyloid pathology in AD cases from that arising in other amyloid-positive diseases, on the basis of a disease-specific pattern of tracer impaired cerebral blood flow (CBF) or energy metabolism. Even more importantly in amyloid-negative cases, further differential diagnoses can be informed by depiction of the hypometabolic/hypoperfusion pattern.

As such, combining amyloid PET with FDG PET or perfusion SPECT delivers complementary information, which helps to improve accuracy of AD diagnosis, and the specification of disease progression (Ossenkoppele et al., 2013). In this regard, it seems relevant that several recent studies have shown comparable reductions of early-phase amyloid PET tracer uptake and metabolic deficits in PET using FDG (Meyer et al., 2011, Rostomian et al., 2011, Hsiao et al., 2012, Tiepolt et al., 2016). This concordance arises from the nature of lipophilic radiotracers such as FBB for amyloid PET and [^99m^Tc]-HMPAO for perfusion SPECT. In general, these lipophilic tracers have a high first-pass influx rate (K_1_) (Dishino et al., 1983), which correlates with the regional CBF due to the high extraction fraction (K_1_/CBF for [^11^C]PiB: 77%, (Blomquist et al., 2008)), and (due to the phenomenon of flow-metabolism coupling), also with the metabolic rate for glucose metabolism (Silverman, 2004, Nihashi et al., 2007, Herholz, 2011). Thus, early-phase PET images with lipophilic tracers can serve as a surrogate for metabolism.

The aim of the present study was to investigate the comparability of early-phase FBB PET, as a depiction of a perfusion-like image, to regional glucose metabolism in FDG PET images, both of which are impaired in patients with dementia. Therefore, we performed relative quantitative cross-analyses as well as visual cross-assessments of early-phase FBB and conventional FDG PET acquisitions, which were acquired in a clinical setting of patients with suspicion of a neurodegenerative dementia disorder.

## 2 Methods

### 2.1 Study design and patient enrollment

All subjects were recruited by the Klinikum der Universität München, the study protocol was approved by the local institutional review board and complied with the declaration of Helsinki. All patients gave their written informed consent and were scanned in a clinical setting at the Department of Nuclear Medicine. The primary objective of the prospective study is the clinical utility of FBB-PET (N = 93 subjects), and in a subset of 33 patients early-phase FBB acquisitions could be performed. All of these included subjects had an additional FDG PET investigation, with < 12 months between FBB and FDG PET.

### 2.2 Radiosynthesis

Radiosynthesis of FBB was performed as described previously (Patt et al., 2010), employing an automated synthesis module (Eckert & Ziegler, Berlin, Germany). Radiochemical purity was > 99% and specific activity was 7.3 × 10^5^ ± 3.4 × 10^5^ GBq mmol^− 1^ at the end of synthesis.

### 2.3 PET imaging

#### 2.3.1 FBB PET acquisition

FBB PET images were acquired in 3D mode on a GE Discovery 690 PET/CT scanner. For those with early recordings, a dynamic emission recording lasting 10 min (10 × 60 s frames) was initiated immediately upon intravenous injection of 300 ± 5 MBq FBB, whereas late static recordings were recorded from 90 min to 110 min p.i. (4 × 300 s) (Barthel et al., 2011). A low-dose CT scan was performed just prior to the static acquisition for attenuation correction of both PET emission recordings. PET data were reconstructed iteratively into a pair of summed early-phase FBB images (0–5 min p.i. (FBB_0–5_) and 0–10 min p.i. (FBB_0–10_)) and one late-phase FBB image (90–110 min p.i. (FBB_90–110_)).

#### 2.3.2 FDG PET acquisition

FDG PET images were acquired using a 3-dimensional GE Discovery 690 PET/CT scanner or a Siemens ECAT EXACT HR + PET scanner. All patients fasted for at least 6 h prior to scanning, and had a maximum plasma glucose level of 120 mg/dl at time of [^18^F]-FDG administration. A dose of 140 ± 7 MBq [^18^F]-FDG was injected intravenously in resting conditions, in a room with dimmed light and low noise level. A static emission frame was acquired from 30 min to 45 min p.i. for the GE Discovery 690 PET/CT, or from 30 to 60 min p.i. for the Siemens ECAT EXACT HR + PET scanner. A low-dose CT scan or a transmission scan with external ^68^Ge-sources was performed prior to the static acquisition and was used for attenuation correction. PET data were reconstructed iteratively (GE Discovery 690 PET/CT, voxel size 2.34 × 2.34 × 3.27 mm, 3D recon with a 4.5 mm Gaussian post filter) or with filtered backprojection (Siemens ECAT EXACT HR + PET, voxel-size 2.03 × 2.03 × 2.42 mm with a 2.42 mm Hann filter). This resulted in datasets with comparable resolution (Joshi et al., 2009).

### 2.4 Image processing

#### 2.4.1 Template generation

For spatial normalization, early-phase FBB (FBB_0–5_, FBB_0–10_) uptake templates and a FDG template were created using the PMOD software (version 3.5, PMOD Technologies Ltd., Zurich, Switzerland). First, individual PET images (FBB_0–5_, FBB_0–10_ and FDG) from 16 randomly selected subjects were rigidly matched to the corresponding individual MR image (T1-weighted). The individual MR images were spatially normalized to a Montreal Neurological Institute (MNI) T1w MRI template, and the individual MR-MR transformation parameters were saved. Consecutively the coregistered PET images were spatially normalized to the MNI template using the individual transformation parameters, scaled to global mean, and smoothed with an 8 mm Gaussian filter. Finally PET templates were generated by calculating the mean of all normalized PET counts in FBB_0–5_, FBB_0–10_ and FDG PET, as previously described (Meyer et al., 1999, Hsiao et al., 2013).

#### 2.4.2 Data processing

All pairs of early-phase FBB images and all FDG images were spatially normalized to the different PET MNI space templates. A total of 83 grey matter volumes of interest (VOIs) predefined in the Hammers atlas (Hammers et al., 2003) were applied to the spatially normalized early-phase amyloid and FDG PET images. Data from the 83 grey matter VOIs were combined resulting in the following cortical target brain regions: frontal, sensorimotor, occipital, temporo-lateral, parietal, posterior cingulate/precuneal cortex, as well as whole brain, separately for the right and left hemispheres. As reference regions for activity normalization, we used whole cerebellum (CBL) or whole brain (= global mean; GLM) including CBL. For relative quantitative analysis, regional standardized uptake value ratios (SUVR) were calculated for each cortical brain VOI, with scaling for either CBL or GLM.

### 2.5 Image analysis

#### 2.5.1 Late-phase FBB PET

Late-phase FBB images were visually assessed by three independent experts in Nuclear Medicine. Patients with significantly increased cortical FBB uptake in at least one cortical region were judged as amyloid-positive according to common diagnostic criteria. A conflicting result between readers in one case was resolved by a majority decision.

#### 2.5.2 Relative quantitative cross-correlation of early-phase [^18^F]-florbetaben PET and FDG PET

Regional initial amyloid uptake and glucose metabolism were assessed relative quantitatively on a VOI base by comparing the regional SUVRs of early-phase FBB PET recordings to the corresponding regional SUVRs from FDG PET. To identify the preferable reference region, we compared correlation coefficients of VOI-based results between early-phase FBB and FDG images, using CBL or GLM for normalization of uptake. Similarly, to identify the better of the two time frames for early-phase amyloid PET imaging, we calculated correlation coefficients for VOI results using FBB_0–5_ or FBB_0–10_ images. In both cases, the preferred reference region or time frame was the one giving the higher correlation coefficients.

#### 2.5.3 Visual analysis of stereotactic surface projections of early-phase FBB and FDG PET

For visual interpretation of early-phase FBB PET (FBB_0–5_) and FDG PET images, three-dimensional stereotactic surface projections (3D-SSP) (Minoshima et al., 1995) were generated using the software Neurostat (Department of Radiology, University of Washington, Seattle, WA, U.S.A.). Three independent experts in Nuclear Medicine visually assessed the 3D-SSP images using tracer uptake and *Z*-score maps (with GLM reference for scaling). Voxel-wise Z-scores were calculated in Neurostat by comparing the individual tracer uptake (FBB_0–5_ and FDG) to historical FDG PET scans from a healthy age-matched cohort (N = 18). For visual analysis, the GLM normalization for FBB PET was chosen because it imparted the visually best resemblance to the corresponding FDG image which was additionally supported by relative quantitative results. All readers were blinded to any identifying and clinical information. All 3D-SSP images (FBB_0–5_ and FDG PET) were uploaded in a random sequence, and readers were not informed of the kind of scan (FBB_0–5_ or FDG). Regional abnormalities (hypoperfusion/hypometabolism) in FBB_0–5_ and FDG images were graded as not relevant = 0, low = 1, moderate = 2 and severe = 3 in the following regions: frontal right and left, temporo-lateral right and left, parietal right and left, posterior cingulate/precuneus for both hemispheres. A PET diagnosis was provided by four-item judgement of the most likely of the following diagnoses: 1. low/moderate hypoperfusion/hypometabolism, that is suspicious of a beginning neurodegenerative disease (e.g. exclusive hypoperfusion/hypometabolism in posterior cingulate cortex), 2. AD, 3. frontotemporal lobar degeneration (FTLD) or 4. non-AD/FTLD including rare neurodegenerative diseases such as corticobasal syndrome, including hypoperfusion/hypometabolism that is not specific for a neurodegenerative disease pattern (e.g. changes due to (minor) strokes in terms of vascular dementia) and also including no relevant hypoperfusion/hypometabolism.

### 2.6 Statistical analysis

Group correlations of regional SUVRs between early-phase FBB and FDG images were evaluated using Pearson's correlation test. For visual analysis, the intra-reader correlations between hypoperfusion in early-phase FBB and hypometabolism in FDG images were calculated by Spearman's rank correlation coefficient (R_s_). For specification of the most likely PET diagnosis, intra-reader agreement between early-phase FBB and FDG was calculated using Cohen's Kappa. A significance level of p < 0.05 was applied in all analyses. All statistical tests were performed using SPSS 22.0.

## 3 Results

### 3.1 Demographics

A total of 33 subjects (19 male) were included in the study. The group consisted of 11 subjects with a clinical diagnosis of mild cognitive impairment (MCI) and 22 demented subjects with different clinical presentations: 11 of these cases had a most likely diagnosis of AD, four were likely suffering from FTLD, single cases of primary progressive aphasia or corticobasal degeneration and five cases with ambiguous clinical and biomarker presentation. The mean age was 68 ± 11 years. 18 of 33 late FBB PETs were visually classified as amyloid-positive (9 male; mean age 69 ± 9 years), 15 of 33 as amyloid-negative (10 male; mean age 66 ± 13 years). The mean ± SD time period between FBB and FDG was 2.7 ± 3.4 months (Table 1**).**

**Table 1:** Demographics of the study population.

Study Groups	N	Age (y ± SD)	Gender (%-m/%-f)	Mean difference between FDG and FBB PET (in month)
All subjects	33	67.5 ± 11.0	58/42	2.7 ± 3.4
Amyloid-positive	18	69.1 ± 8.7	50/50	3.0 ± 4.5
Amyloid-negative	15	66.3 ± 12.8	67/33	2.4 ± 3.4

### 3.2 VOI-based comparison of early-phase FBB and FDG PET

**Fig. 1:**
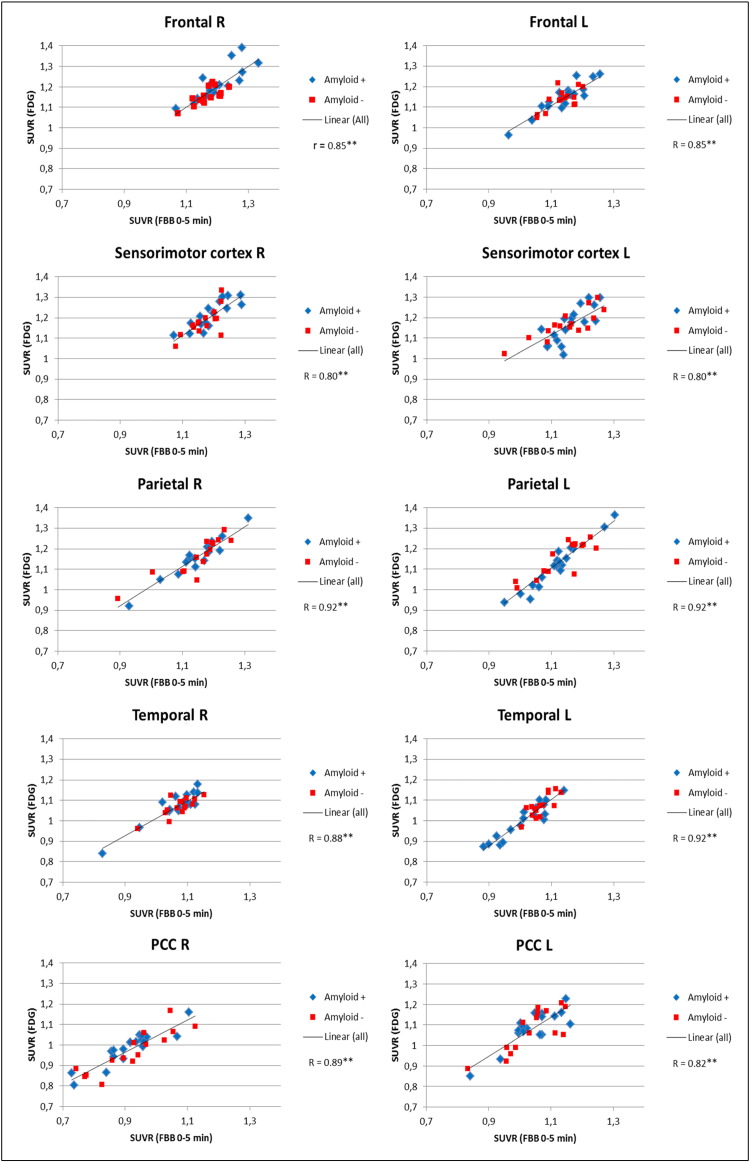
Correlation charts of early-phase FBB_0–5_ and FDG SUVRs (global mean normalization). R: right; L: left; PCC: posterior cingulate cortex; **p < 0.01.

Correlation plots for FBB_0–5_ versus FDG PET with GLM normalization are shown in Fig. 1. Regional SUVRs and correlation coefficients determined by comparing regional FBB_0–5_ and FBB_0–10_ with FDG SUVRs (CBL and GLM normalization) are shown in Table 2. All cortical brain regions showed highly significant correlations irrespective of the early-phase FBB time frame or the particular reference region (p < 0.0001). The least correlation was found in the left frontal and right sensorimotor region (R_0–5/CBL_ = 0.59) and the highest in the left and right parietal region, the left temporo-lateral region (R_0–5/GLM_ = 0.92) as well as the right parietal region (R_0–10/GLM_ = 0.92). Overall, the highest correlation values were found for a GLM normalization irrespective of the particular FBB time frame, for which the mean correlations among regions were R_0–5/GLM_ = 0.86 ± 0.05 and R_0–10/GLM_ = 0.86 ± 0.05. In comparison, the CBL normalization gave strong, but significantly lower correlations (p < 0.001; paired *t*-test) between FBB and FDG SUVRs (R_0–5/CBL_ = 0.75 ± 0.10 and R_0–10/CBL_ = 0.76 ± 0.10) (Table 2).

**Table 2:** Regional SUVRs and correlation coefficients of early-phase FBB and FDG with cerebellar and global mean normalization. R: right; L: left; PCC: posterior cingulate cortex; **p < 0.01.

All subjects	Global mean normalization					Cerebellum normalization				
Region	Early-phase [^18^F]-florbetaben PET				[^18^F]-FDG PET	Early-phase [^18^F]-florbetaben PET				[^18^F]-FDG PET
	0–5 min (SUVR ± SD)	R	0–10 min (SUVR ± SD)	R	(SUVR ± SD)	0–5 min (SUVR ± SD)	R	0–10 min (SUVR ± SD)	R	(SUVR ± SD)
Frontal R	1.19 ± 0.06	0.82**	1.18 ± 0.05	0.81**	1.19 ± 0.07	0.99 ± 0.07	0.65**	1.00 ± 0.06	0.69**	1.09 ± 0.09
Frontal L	1.14 ± 0.06	0.85**	1.13 ± 0.06	0.83**	1.14 ± 0.06	0.95 ± 0.06	0.59**	0.96 ± 0.05	0.60**	1.05 ± 0.08
Sensorimotor R	1.18 ± 0.05	0.80**	1.17 ± 0.05	0.79**	1.20 ± 0.07	0.98 ± 0.07	0.59**	0.99 ± 0.06	0.60**	1.10 ± 0.08
Sensorimotor L	1.16 ± 0.07	0.77**	1.14 ± 0.07	0.77**	1.16 ± 0.08	0.96 ± 0.08	0.66**	0.97 ± 0.07	0.65**	1.07 ± 0.10
Occipital R	1.30 ± 0.06	0.81**	1.28 ± 0.06	0.82**	1.26 ± 0.07	1.08 ± 0.09	0.70**	1.09 ± 0.07	0.73**	1.16 ± 0.08
Occipital L	1.30 ± 0.08	0.90**	1.28 ± 0.08	0.90**	1.27 ± 0.10	1.08 ± 0.09	0.78**	1.09 ± 0.07	0.78**	1.16 ± 0.10
Parietal R	1.15 ± 0.08	0.92**	1.14 ± 0.08	0.92**	1.16 ± 0.09	0.96 ± 0.10	0.83**	0.98 ± 0.09	0.85**	1.07 ± 0.12
Parietal L	1.12 ± 0.08	0.92**	1.12 ± 0.07	0.90**	1.13 ± 0.10	0.94 ± 0.09	0.82**	0.96 ± 0.08	0.82**	1.04 ± 0.12
PCC R	0.92 ± 0.10	0.89**	0.95 ± 0.08	0.91**	0.98 ± 0.09	0.76 ± 0.10	0.86**	0.81 ± 0.09	0.88**	0.90 ± 0.11
PCC L	1.04 ± 0.08	0.82**	1.05 ± 0.06	0.88**	1.08 ± 0.09	0.86 ± 0.08	0.76**	0.90 ± 0.07	0.78**	0.99 ± 0.11
Temporo-lateral R	1.07 ± 0.06	0.88**	1.08 ± 0.06	0.87**	1.07 ± 0.06	0.89 ± 0.08	0.85**	0.92 ± 0.07	0.88**	0.98 ± 0.07
Temporo-lateral L	1.04 ± 0.06	0.92**	1.05 ± 0.05	0.91**	1.03 ± 0.08	0.87 ± 0.08	0.85**	0.89 ± 0.06	0.86**	0.95 ± 0.09
Whole R	1.14 ± 0.04	0.86**	1.14 ± 0.04	0.87**	1.14 ± 0.04	0.95 ± 0.07	0.70**	0.97 ± 0.06	0.74**	1.05 ± 0.07
Whole L	1.12 ± 0.04	0.87**	1.12 ± 0.03	0.83**	1.12 ± 0.05	0.93 ± 0.06	0.70**	0.95 ± 0.05	0.65**	1.03 ± 0.07

To determine the preferable time frame for initial FBB uptake, we compared the correlation values between FBB_0–5_ and FBB_0–10_ uptake and FDG results. Using the GLM reference, there was no significant difference in the correlation coefficients (mean R_0–5/GLM_ = 0.86 vs. R_0–10/GLM_ = 0.86; p = ns; paired *t*-test). In contrast, CBL normalization gave slightly stronger correlations for a time frame of 0–10 min (mean R_0–5/CBL_ = 0.75 vs. mean R_0–10/CBL_ = 0.76; *p* < 0.05; paired t-test).

All relative quantitative analyses were repeated after splitting the cohort into an amyloid-positive (n = 18) and amyloid-negative (n = 15) subgroup. The corresponding SUVRs and correlation coefficients are shown in Table 3A and B. All brain regions in the amyloid-positive cohort as well as nearly all in the amyloid-negative cohort (with exception of right sensorimotor cortex with CBL normalization and FBB_0–10_ (R_0–10/CBL_ = 0.41)) showed significant regional correlation values between early-phase FBB results and FDG images (with either CBL or GLM normalization). Significantly higher correlations were observed in the amyloid-positive group (e.g. mean R_0–5/GLM_ = 0.90 (for amyloid-positive) versus mean R_0–5/GLM_ = 0.79 (for amyloid-negative), p < 0.001). For the entire cohort, the regional SUVRs with a GLM normalization showed better correlations between early-phase FBB and FDG than did SUVRs with CBL normalization; there was no difference between correlations for FBB_0–5_ and FBB_0–10_ results when using the GLM normalization, whereas CBL normalization gave better correlations for FBB_0–10_ in the amyloid-positive subgroup and for FBB_0–5_ in the amyloid-negative subgroup (Table 4).

**Table 3:** Regional SUVRs and correlation coefficients of early-phase FBB and FDG of amyloid-positive (A) and amyloid-negative (B) subjects. R: right; L: left; PCC: posterior cingulate cortex; **p < 0.01.

Region	Global mean normalization					Cerebellum normalization				
	Early-phase [^18^F]-florbetaben PET				[^18^F]-FDG PET	Early-phase [^18^F]-florbetaben PET				[^18^F]-FDG PET
	0–5 min (SUVR ± SD)	R	0–10 min (SUVR ± SD)	R	(SUVR ± SD)	0–5 min (SUVR ± SD)	R	0–10 min (SUVR ± SD)	R	(SUVR ± SD)
Amyloid-positive										
Frontal R	1.20 ± 0.07	0.82**	1.19 ± 0.05	0.82**	1.21 ± 0.08	0.99 ± 0.07	0.64**	1.01 ± 0.06	0.73**	1.11 ± 0.09
Frontal L	1.14 ± 0.07	0.92**	1.14 ± 0.06	0.91**	1.15 ± 0.07	0.94 ± 0.06	0.64**	0.97 ± 0.05	0.71**	1.05 ± 0.09
Sensorimotor R	1.19 ± 0.06	0.84**	1.18 ± 0.05	0.85**	1.21 ± 0.07	0.98 ± 0.08	0.70**	1.00 ± 0.06	0.75**	1.11 ± 0.08
Sensorimotor L	1.16 ± 0.06	0.78**	1.15 ± 0.05	0.77**	1.16 ± 0.09	0.96 ± 0.08	0.68**	0.98 ± 0.06	0.71**	1.06 ± 0.10
Occipital R	1.29 ± 0.07	0.90**	1.27 ± 0.06	0.90**	1.27 ± 0.07	1.07 ± 0.08	0.70**	1.08 ± 0.07	0.79**	1.16 ± 0.08
Occipital L	1.29 ± 0.09	0.93**	1.27 ± 0.08	0.93**	1.26 ± 0.11	1.07 ± 0.10	0.80**	1.08 ± 0.07	0.85**	1.15 ± 0.10
Parietal R	1.15 ± 0.08	0.96**	1.15 ± 0.08	0.95**	1.16 ± 0.09	0.95 ± 0.10	0.87**	0.98 ± 0.09	0.92**	1.06 ± 0.11
Parietal L	1.12 ± 0.09	0.97**	1.12 ± 0.08	0.95**	1.12 ± 0.11	0.92 ± 0.09	0.87**	0.95 ± 0.07	0.92**	1.02 ± 0.13
PCC R	0.91 ± 0.10	0.92**	0.95 ± 0.07	0.93**	0.98 ± 0.08	0.75 ± 0.08	0.85**	0.81 ± 0.07	0.83**	0.90 ± 0.09
PCC L	1.04 ± 0.08	0.84**	1.06 ± 0.06	0.90**	1.09 ± 0.09	0.86 ± 0.06	0.78**	0.90 ± 0.05	0.75**	0.99 ± 0.09
Temporo-lateral R	1.06 ± 0.08	0.92**	1.07 ± 0.07	0.91**	1.07 ± 0.07	0.88 ± 0.09	0.84**	0.91 ± 0.08	0.92**	0.98 ± 0.08
Temporo-lateral L	1.01 ± 0.07	0.94**	1.03 ± 0.06	0.93**	1.00 ± 0.08	0.84 ± 0.08	0.84**	0.87 ± 0.06	0.89**	0.92 ± 0.10
Whole R	1.14 ± 0.04	0.93**	1.14 ± 0.04	0.95**	1.15 ± 0.04	0.95 ± 0.07	0.72**	0.97 ± 0.06	0.83**	1.05 ± 0.07
Whole L	1.11 ± 0.04	0.91**	1.11 ± 0.04	0.89**	1.11 ± 0.05	0.92 ± 0.06	0.67**	0.94 ± 0.04	0.75**	1.01 ± 0.07

Amyloid-negative										
Frontal R	1.17 ± 0.04	0.74**	1.16 ± 0.04	0.67**	1.15 ± 0.04	0.98 ± 0.07	0.68**	0.99 ± 0.07	0.61**	1.07 ± 0.08
Frontal L	1.13 ± 0.05	0.68**	1.13 ± 0.05	0.63**	1.14 ± 0.05	0.95 ± 0.06	0.52*	0.96 ± 0.06	0.46*	1.05 ± 0.08
Sensorimotor R	1.17 ± 0.05	0.71**	1.16 ± 0.04	0.69**	1.18 ± 0.07	0.98 ± 0.06	0.45*	0.99 ± 0.06	0.41	1.09 ± 0.09
Sensorimotor L	1.15 ± 0.09	0.84**	1.14 ± 0.08	0.85**	1.17 ± 0.07	0.97 ± 0.08	0.65**	0.97 ± 0.08	0.63**	1.08 ± 0.09
Occipital R	1.31 ± 0.05	0.72**	1.29 ± 0.05	0.74**	1.26 ± 0.07	1.01 ± 0.07	0.72**	1.10 ± 0.07	0.67**	1.16 ± 0.09
Occipital L	1.31 ± 0.07	0.83**	1.29 ± 0.06	0.83**	1.27 ± 0.09	1.10 ± 0.07	0.75**	1.10 ± 0.06	0.68**	1.18 ± 0.10
Parietal R	1.15 ± 0.09	0.88**	1.14 ± 0.08	0.89**	1.16 ± 0.09	0.97 ± 0.11	0.80**	0.97 ± 0.08	0.79**	1.07 ± 0.13
Parietal L	1.13 ± 0.08	0.85**	1.13 ± 0.07	0.84**	1.15 ± 0.09	0.96 ± 0.10	0.74**	0.96 ± 0.09	0.72**	1.07 ± 0.12
PCC R	0.92 ± 0.11	0.89**	0.95 ± 0.10	0.90**	0.97 ± 0.10	0.78 ± 0.12	0.88**	0.81 ± 0.11	0.90**	0.90 ± 0.13
PCC L	1.04 ± 0.09	0.80**	1.05 ± 0.08	0.86**	1.07 ± 0.10	0.87 ± 0.10	0.76**	0.89 ± 0.09	0.79**	0.99 ± 0.13
Temporo-lateral R	1.07 ± 0.05	0.78**	1.08 ± 0.05	0.77**	1.07 ± 0.05	0.90 ± 0.08	0.87**	0.92 ± 0.08	0.85**	0.99 ± 0.06
Temporo-lateral L	1.07 ± 0.04	0.79**	1.07 ± 0.03	0.78**	1.07 ± 0.05	0.90 ± 0.06	0.84**	0.91 ± 0.06	0.79**	0.99 ± 0.07
Whole R	1.14 ± 0.03	0.75**	1.13 ± 0.31	0.73**	1.13 ± 0.03	0.96 ± 0.07	0.71**	0.97 ± 0.06	0.66**	1.05 ± 0.08
Whole L	1.13 ± 0.03	0.78**	1.12 ± 0.31	0.73**	1.13 ± 0.04	0.95 ± 0.06	0.63**	0.96 ± 0.05	0.58*	1.04 ± 0.07

**Table 4:** Mean correlation coefficients of early-phase FBB vs. FDG. GLM: global mean normalization, CBL: cerebellum normalization; *p < 0.05; **p < 0.01.

Parameters (reference region, time frame)	Mean R between early-phase FBB and FDG		
	All	Amyloid-positive	Amyloid-negative
GLM, 0–5 min	0.86 ± 0.05	0.90 ± 0.06	0.79 ± 0.07
GLM, 0–10 min	0.86 ± 0.05	0.90 ± 0.06	0.79 ± 0.09
CBL, 0–5 min	0.75 ± 0.10	0.77 ± 0.09	0.72 ± 0.13
CBL, 0–10 min	0.76 ± 0.10	0.81 ± 0.08	0.69 ± 0.15

### 3.3 Visual 3D-SSP comparison of early-phase FBB and FDG

**Fig. 2:**
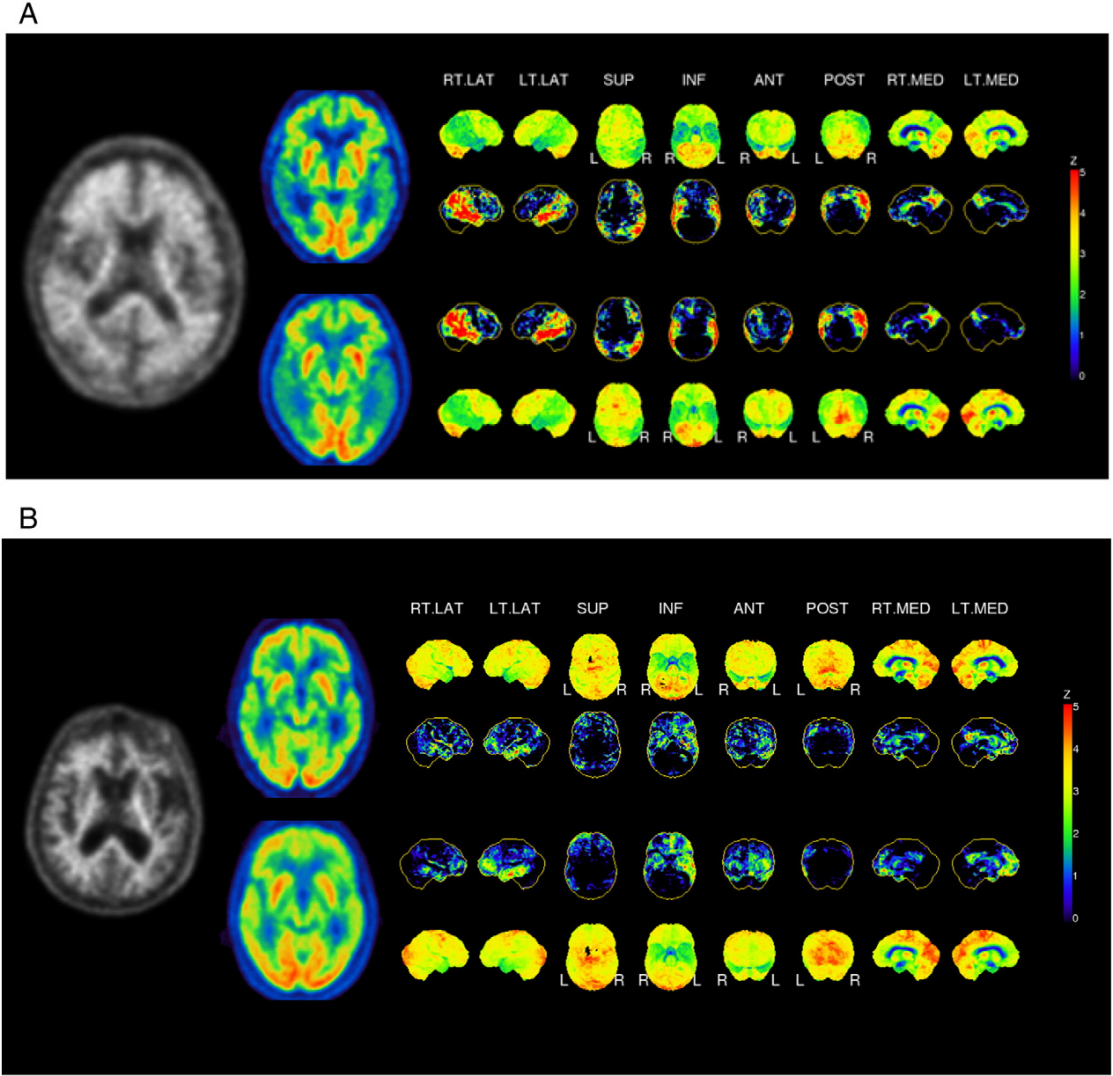
3D-SSP images of a 79 year old male person with clinical presentation of AD (A) and an 81 year old male person with clinical presentation of FTLD (B). Normalized count and *Z*-maps for FDG (upper row) and FBB_0–5_. The map depicts areas with less uptake compared to normal controls. R: right; L: left; PCC: posterior cingulate cortex

After identifying the optimal time frame and reference region, visual assessment was performed by evaluating 3D-SSP images of early-phase FBB_0–5_ and FDG images of tracer uptake and *Z*-scores (with GLM normalization). Fig. 2A shows 3D-SSP images for a 79 year old male with clinical presentation of AD, Fig. 2B an 81 year old male with clinical presentation of FTLD. The regional pattern of the perfusion surrogate in early-phase FBB_0–5_ images resembles the FDG uptake pattern, as can be seen both in an amyloid-positive and amyloid-negative case.

**Fig. 3:**
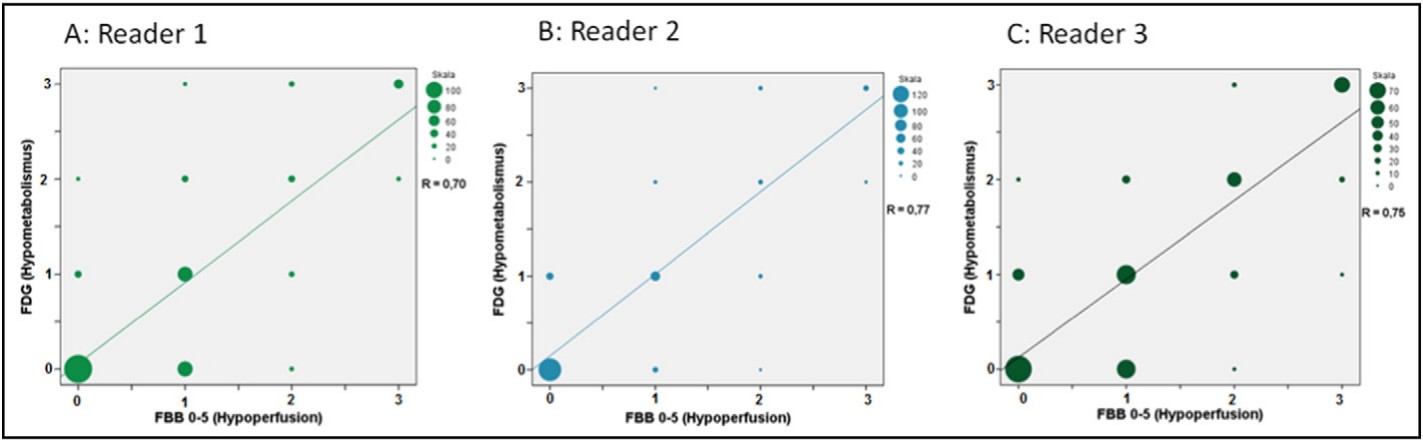
Correlation of visual scores for hypoperfusion/hypometabolism severity by three readers in early-phase FBB_0–5_ and FDG images.

The visual assessment of all target regions in all 33 patients showed a Spearman's rank correlation coefficient between FBB_0–5_ and FDG of R_s_ = 0.70 for reader 1, R_s_ = 0.77 for reader 2 and R_s_ = 0.75 for reader 3 (mean R_s_ = 0.74) (Fig. 3). Regarding Fig. 3 reader 1 and 3 have a considerable number of assessed regions with early FBB = 1 and FDG = 0. This could lead to the interpretation that early FBB images may demonstrate more severe hypoperfusion. In relative quantitative analysis this does not prove true (SUVR(FBB_0–5)_ vs. SUVR (FDG), p = ns).

**Fig. 4:**
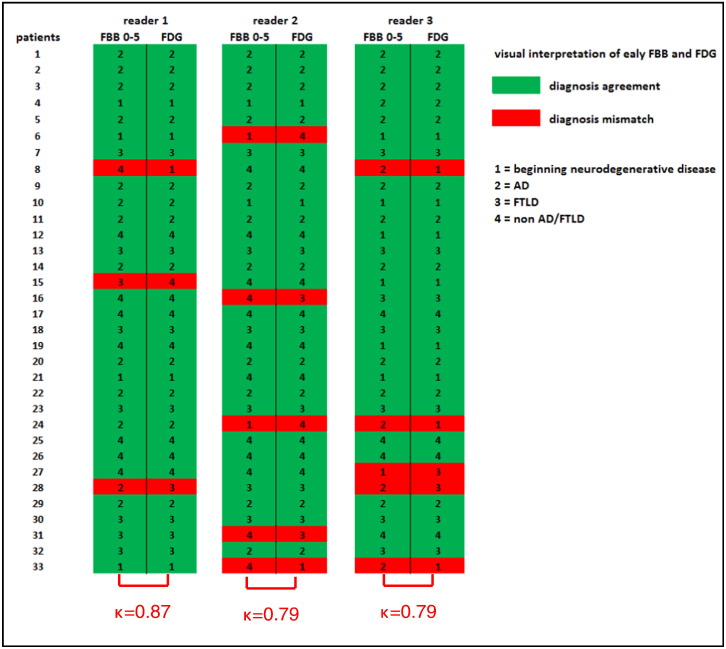
Agreement and mismatch of individual PET diagnosis in visual interpretation of early-phase FBB_0–5_ and FDG 3D–SSP images. AD: Alzheimer's disease; FTLD: Frontotemporal lobar degeneration.

Specifying the most likely diagnosis using 3D-SSP images of early-phase FBB and FDG PET, reader 1 showed an overlapping diagnosis in 30 of 33 patients corresponding to an intra-reader agreement of κ = 0.87. Readers 2 and 3 showed an overlap in 28 of 33 patients, corresponding to intra-reader agreements of κ = 0.79 (mean κ = 0.82, p < 0.0001). There were a total of 13 cases (in 9 patients; 5 amyloid-negative, 4 amyloid-positive) of a mismatched PET diagnosis in 99 comparisons performed by the three readers. In four of these 13 cases, the discrepant classification was non-AD/FTLD versus beginning neurodegenerative disease, another four cases beginning neurodegenerative disease versus AD or FTLD, in three cases there were diagnoses of non-AD/FTLD versus AD or FTLD, and in two cases AD versus FTLD (Fig. 4**).**

## 4 Discussion

Whereas previous studies of this type have had their main focus on VOI-based or voxel-based statistical analysis of early-phase amyloid PET (Meyer et al., 2011, Hsiao et al., 2012), it was our aim to investigate the clinical use of FBB PET by additional visual interpretation of early-phase FBB acquisitions. Our results demonstrate the strong visual and relative quantitative correlations of initial FBB uptake with FDG images, irrespective of the amyloid status demonstrated by the late-phase FBB scans. Thus, early-phase FBB acquisitions, which are highly weighted to cerebral perfusion, seem to be a valid surrogate marker for synaptic and metabolic dysfunction. A brief additional FBB recording in the initial minutes after tracer injection afford supplemental information about neuronal activity, which we believe can ultimately obviate the need for an FDG PET scan. For patients, this means less exposure to radiation and sparing of an additional visit to the clinic. Not to be disregarded, the greater comfort of persons investigated for a neurodegenerative disease might well lead to improved patient and caregiver compliance.

The results of relative quantitative, VOI-based statistical analysis show a strong correlation of regional tracer uptake (SUVR) in all investigated cortical brain regions between initial FBB uptake and FDG PET. This is in perfect agreement with previous studies detecting high correlations between amyloid ([^11^C]-PIB and [^18^F]-AV45) R_1_ images derived from the simplified reference tissue analysis (where R_1_ is an index of relative CBF), as well as early time frame images of [^11^*C*]PiB or FBB and FDG PET (Meyer et al., 2011, Rostomian et al., 2011, Hsiao et al., 2012, Tiepolt et al., 2016). The study of Tiepolt et al. investigating a mixed cohort of early [^11^C]-PIB and early FBB scans found regional correlation values ranging from r = 0.609 to r = 0.788 (using a time frame of 1–9 min and CBL as the reference region). Using comparable parameters with a time frame of 0–10 min and CBL as the reference region we found correlation values ranging from r = 0.60 to r = 0.88. The slightly lower correlations in the work of Tiepolt et al. may be explained by the smaller sample size and the different tracers, since they showed stronger correlations between early FBB and FDG compared to early [^11^C]-PIB and FDG.

After splitting the whole cohort into amyloid-positive and amyloid-negative subgroups, there emerged even higher correlation values in those with amyloidosis, irrespective of the reference region or the time frame (0–5 or 0–10 min). This may be explained by a greater prevalence of neurodegenerative cases (especially AD) in conjunction with rather more severe hypoperfusion/hypometabolism in the amyloid-positive group, i.e. greater dynamic range, which leads to better separation. On the other hand, the amyloid-negative subgroup consisted of fewer cases with severe hypoperfusion/hypometabolism, manifesting in less defects in the early-phase FBB images. Besides, the higher correlation values in the amyloid-positive subgroup may as well be influenced by the larger cohort compared to the amyloid-negative subgroup (n = 18 vs. n = 15). That the amyloid-positive cases showed excellent correlations between early-phase FBB PET and metabolism in FDG PET lends further support to the contention that present cortical amyloid pathology need not have a relevant effect on the extent of perfusion/metabolism coupling (Spehl et al., 2015), although this may still require additional validation. We found best correlations between the two PET measures for GLM normalization, and slightly lower correlations for CBL normalization. CBL VOIs are typically used as the preferred reference region for calculation of SUVRs because of low or absent amyloid plaque burden in the cerebellar cortex of AD patients (Svedberg et al., 2009, Barthel et al., 2011). Especially for longitudinal evaluations of late amyloid PET, it is self-evident that the reference region should not itself be affected by amyloid deposition. In the present context, images of initial FBB uptake do not reflect amyloid burden per se, but are rather a surrogate of CBF, due to the very first pass high extraction of FBB and other lipophilic tracers. We note that cerebellar perfusion can itself be affected by crossed cerebellar diaschisis in neurodegenerative diseases, which might propagate to bias in normalized SUV calculations. While there is generally good coupling between CBF and metabolism, others have shown that the CBL is relatively hyperperfused compared to its rate of glucose metabolism (Gur et al., 2009). As such, the CBL need not be considered entirely privileged with respect to perfusion changes in neurodegenerative diseases. However, it remains unclear if this is the cause for our present finding of lower correlation values when using CBL rather than GLM normalization. Further studies, perhaps using data-driven methods (Dukart et al., 2013), might identify an even better reference region for SUVR-based analysis of early-phase amyloid PET.

To define the FBB time frame with highest correlation to metabolism we compared early-phase (FBB_0–5_ and FBB_0–10_) FBB acquisitions to FDG PET. Given the GLM normalized SUVRs, we observed no difference in the correlations resulting from the two early frame dimensions. In contrast, when using the CBL as the normalization reference region, there were some inconsistent results. Whereas the time frame of 0–10 min seemed to provide better results in the amyloid-positive subgroup, the time frame of 0–5 min was superior in the amyloid-negative subgroup, which is likely to have less severe perfusion and metabolism defects. Previous studies have compared a range of early-phase amyloid PET time frames with respect to correlations with FDG PET using voxel-wise or regional analyses. One study indicated a superior time window of 1–8 min p.i. for [^11^C]-PIB (Rostomian et al., 2011), whereas another study found best agreement with 1–6 min p.i. for the case of [^11^*C*]AV-45 (Hsiao et al., 2012). There may be no universally applicable time frame for such purposes, since the time interval for optimal perfusion weighting will depend on the particular amyloid tracer, administration technique, and perhaps also the extent of degenerative changes (as suggested by our somewhat different results for amyloid-positive and -negative subgroups). Taken together, we found no significant difference between 5 and 10 min acquisitions and therefore suggest for the latest generation of PET scanners, for the sake of patient comfort and economy, the shorter acquisition protocol. In case of older and less sensitive PET scanners a longer acquisition time might be useful in order to ensure sufficient count statistics.

In the second part of our study we visually analyzed the comparability of initial FBB uptake to FDG 3D-SSP images using tracer count rates and *Z*-score maps, for which we employed maps with GLM normalization. The decision was based on the visually-judged greater resemblance of the resultant images to the corresponding FDG images. In contrast, CBL, thalamus and pons normalizations simulated severe hypoperfusion of cortical areas in the 3D-SSP images of initial FBB uptake. This might arise from a relative hyperperfusion compared to metabolism in subcortical regions, as seen in a previous study (Gur et al., 2009), and as supported by the high perfusion-to-metabolism ratio reported for CBL and thalamus (Hsiao et al., 2012). To investigate the similarity of early-phase amyloid PET and FDG PET regarding the occurrence of hypometabolism/hypoperfusion, we had three independent readers grading the severity of hypometabolism/hypoperfusion (levels 0–3) in four target regions in both hemispheres. Using the Spearman's rank correlation test, all three readers returned highly significant correlations, representing a very good intra-reader agreement. These findings underline the highly significant results that can be derived from relative quantitative analyses, and demonstrate that comparability between these methods is not only limited to relative quantitative PET, but also for visual analyses of initial FBB uptake and FDG images. Consistently to our findings the study of Tiepolt et al. showed concordant regional hypoperfusion/hypometabolism scores (levels 0–4) in 94.7% of 132 visually scored brain VOIs in 11 early FBB and the corresponding FDG scans (95.5% in 11 early [^11^C]-PIB and FDG scans) (Tiepolt et al., 2016).

For the clinical routine it is not only important that there is comparable intensity of early-phase FBB and FDG uptake, it is also important to make the correct diagnosis on the basis of the individual tracer uptake patterns. Therefore three readers specified the most likely PET diagnosis from among four entities, likely to be encountered in this setting: beginning neurodegenerative disease, AD, FTLD or non-AD/FTLD. Reader 1 performed with almost perfect (κ = 0.87) intra-reader agreement, whereas readers 2 and 3 still had substantial overlap (κ = 0.79) in a blinded reading without any clinical information. It needs to be emphasized that this was an unselected cohort of MCI and otherwise demented patients, with some ambiguous cases with diverse tracer uptake patterns ranging from minimal hypometabolism/hypoperfusion to the uptake pattern characteristic of rare neurodegenerative diseases (e.g. progressive supranuclear palsy). A priori knowledge of the clinical presentation or additional neuro-imaging (MRI/CT) results as it is standard in clinical routine PET imaging, could well facilitate finding the most likely diagnosis, and might have resulted in an even better intra-reader agreement. In summary, the results of visual assessment in the present study demonstrate the comparability of early-phase FBB and FDG 3D-SSP images.

As a limitation of this study the absence of an early-phase FBB healthy control database needs to be mentioned. Thus voxel-wise *Z*-scores of early-phase FBB uptake were calculated by comparing the FBB tracer uptake to FDG PET data from a healthy age-matched cohort. Another limitation is due to the fact that FBB and FDG PET scans were in part recorded on different PET scanners, however the reconstruction methods used we were able to harmonize the scanner resolution as much as possible, making a major impact unlikely.

## 5 Conclusions

The present study demonstrates that the initial [^18^F]-florbetaben uptake correlates both relative quantitatively and visually highly with FDG images, irrespective of the particular amyloid status. Thus, [^18^F]-florbetaben uptake in the first ten minutes post injection yields a perfusion-like image, evidently serving as a valid surrogate marker for synaptic and metabolic dysfunction, otherwise revealed in a separate FDG PET scan. Thus, a two-phase [^18^F]-florbetaben protocol might in the future give unambiguous combined neurodegeneration and amyloid pathology biomarker information, while sparing the patient radiation exposure from an additional FDG PET scan. The optimal relative quantitative analysis of early-phase [^18^*F*]florbetaben acquisitions arises from GLM normalization, with little effect of the particular time frame (0–5 or 0–10 min), favoring the shorter time in up-to-date PET scanners due to patient comfort and economic reasons.

## Conflict of interest

AS and MP received research grants from Bayer Healthcase/Piramal Imaging. HB and OS received consultant and speaker honoraria as well as travel expenses from Bayer Healthcare/Piramal Imaging. PB and AR received speaker honoraria from GE and Piramal Imaging.

## References

[bb0005] Barthel H., Sabri O. (2011). Florbetaben to trace amyloid-beta in the Alzheimer brain by means of PET. J. Alzheimers Dis..

[bb0010] Barthel H., Gertz H.J., Dresel S., Peters O., Bartenstein P., Buerger K., Hiemeyer F., Wittemer-Rump S.M., Seibyl J., Reininger C., Sabri O. (2011). Cerebral amyloid-beta PET with florbetaben ((18)F) in patients with Alzheimer's disease and healthy controls: a multicentre phase 2 diagnostic study. Lancet Neurol..

[bb0015] Blomquist G., Engler H., Nordberg A., Ringheim A., Wall A., Forsberg A., Estrada S., Frandberg P., Antoni G., Langstrom B. (2008). Unidirectional influx and net accumulation of PIB. Open Neuroimaging J..

[bb0020] Braak H., Braak E. (1991). Neuropathological stageing of Alzheimer-related changes. Acta Neuropathol..

[bb0025] Dishino D.D., Welch M.J., Kilbourn M.R., Raichle M.E. (1983). Relationship between lipophilicity and brain extraction of C-11-labeled radiopharmaceuticals. J. Nucl. Med..

[bb0030] Donaghy P., Thomas A.J., O'Brien J.T. (2015). Amyloid PET imaging in Lewy body disorders. Am. J. Geriatr. Psychiatry.

[bb0035] Dukart J., Perneczky R., Forster S., Barthel H., Diehl-Schmid J., Draganski B., Obrig H., Santarnecchi E., Drzezga A., Fellgiebel A., Frackowiak R., Kurz A., Muller K., Sabri O., Schroeter M.L., Yakushev I. (2013). Reference cluster normalization improves detection of frontotemporal lobar degeneration by means of FDG-PET. PLoS One.

[bb0040] Furst A.J., Rabinovici G.D., Rostomian A.H., Steed T., Alkalay A., Racine C., Miller B.L., Jagust W.J. (2012). Cognition, glucose metabolism and amyloid burden in Alzheimer's disease. Neurobiol. Aging.

[bb0045] Gur R.C., Ragland J.D., Reivich M., Greenberg J.H., Alavi A., Gur R.E. (2009). Regional differences in the coupling between resting cerebral blood flow and metabolism may indicate action preparedness as a default state. Cereb. Cortex.

[bb0050] Hammers A., Allom R., Koepp M.J., Free S.L., Myers R., Lemieux L., Mitchell T.N., Brooks D.J., Duncan J.S. (2003). Three-dimensional maximum probability atlas of the human brain, with particular reference to the temporal lobe. Hum. Brain Mapp..

[bb0055] Herholz K. (2011). Perfusion SPECT and FDG-PET. Int. Psychogeriatr..

[bb0060] Hsiao I.T., Huang C.C., Hsieh C.J., Hsu W.C., Wey S.P., Yen T.C., Kung M.P., Lin K.J. (2012). Correlation of early-phase 18F-florbetapir (AV-45/Amyvid) PET images to FDG images: preliminary studies. Eur. J. Nucl. Med. Mol. Imaging.

[bb0065] Hsiao I.T., Huang C.C., Hsieh C.J., Wey S.P., Kung M.P., Yen T.C., Lin K.J. (2013). Perfusion-like template and standardized normalization-based brain image analysis using 18F-florbetapir (AV-45/Amyvid) PET. Eur. J. Nucl. Med. Mol. Imaging.

[bb0070] Joshi A., Koeppe R.A., Fessler J.A. (2009). Reducing between scanner differences in multi-center PET studies. NeuroImage.

[bb0075] Kadir A., Almkvist O., Forsberg A., Wall A., Engler H., Langstrom B., Nordberg A. (2012). Dynamic changes in PET amyloid and FDG imaging at different stages of Alzheimer's disease. Neurobiol. Aging.

[bb0080] Lim Y.Y., Ellis K.A., Pietrzak R.H., Ames D., Darby D., Harrington K., Martins R.N., Masters C.L., Rowe C., Savage G., Szoeke C., Villemagne V.L., Maruff P., A. R. Group (2012). Stronger effect of amyloid load than APOE genotype on cognitive decline in healthy older adults. Neurology.

[bb0085] Lim Y.Y., Maruff P., Pietrzak R.H., Ellis K.A., Darby D., Ames D., Harrington K., Martins R.N., Masters C.L., Szoeke C., Savage G., Villemagne V.L., Rowe C.C., A. R. Group (2014). Abeta and cognitive change: Examining the preclinical and prodromal stages of Alzheimer's disease. Alzheimers Dement..

[bb0090] Meyer J.H., Gunn R.N., Myers R., Grasby P.M. (1999). Assessment of spatial normalization of PET ligand images using ligand-specific templates. NeuroImage.

[bb0095] Meyer P.T., Hellwig S., Amtage F., Rottenburger C., Sahm U., Reuland P., Weber W.A., Hull M. (2011). Dual-biomarker imaging of regional cerebral amyloid load and neuronal activity in dementia with PET and 11C-labeled Pittsburgh compound B. J. Nucl. Med..

[bb0100] Minoshima S., Frey K.A., Koeppe R.A., Foster N.L., Kuhl D.E. (1995). A diagnostic approach in Alzheimer's disease using three-dimensional stereotactic surface projections of fluorine-18-FDG PET. J. Nucl. Med..

[bb0105] Nihashi T., Yatsuya H., Hayasaka K., Kato R., Kawatsu S., Arahata Y., Iwai K., Takeda A., Washimi Y., Yoshimura K., Mizuno K., Kato T., Naganawa S., Ito K. (2007). Direct comparison study between FDG-PET and IMP-SPECT for diagnosing Alzheimer's disease using 3D-SSP analysis in the same patients. Radiat. Med..

[bb0110] Ossenkoppele R., Prins N.D., Pijnenburg Y.A., Lemstra A.W., van der Flier W.M., Adriaanse S.F., Windhorst A.D., Handels R.L., Wolfs C.A., Aalten P., Verhey F.R., Verbeek M.M., van Buchem M.A., Hoekstra O.S., Lammertsma A.A., Scheltens P., van Berckel B.N. (2013). Impact of molecular imaging on the diagnostic process in a memory clinic. Alzheimers Dement..

[bb0115] Patt M., Schildan A., Barthel H., Schultze-Mosgau M.H., Rohde B., Reininger C., Sabri O. (2010). Metabolite analysis of [18*F*]Florbetaben (BAY 94-9172) in human subjects: a substudy within a proof of mechanism clinical trial. J. Radioanal. Nucl. Chem..

[bb0120] Rostomian A.H., Madison C., Rabinovici G.D., Jagust W.J. (2011). Early 11C-PIB frames and 18F-FDG PET measures are comparable: a study validated in a cohort of AD and FTLD patients. J. Nucl. Med..

[bb0125] Shokouhi S., Claassen D., Kang H., Ding Z., Rogers B., Mishra A., Riddle W.R. (2013). Longitudinal progression of cognitive decline correlates with changes in the spatial pattern of brain 18F-FDG PET. J. Nucl. Med..

[bb0130] Silverman D.H. (2004). Brain 18F-FDG PET in the diagnosis of neurodegenerative dementias: comparison with perfusion SPECT and with clinical evaluations lacking nuclear imaging. J. Nucl. Med..

[bb0135] Spehl T.S., Hellwig S., Frings L., Bormann T., Meyer P.T. (2015). Einfluss der zerebralen Beta-Amyloid Pathologie auf R1-basierte Bildgebung zerebraler Perfusion mittels C-11-PIB-PET: Hinweise auf eine Störung der Fluss/Metabolismus-Kopplung?. Nucl. Med..

[bb0140] Svedberg M.M., Hall H., Hellstrom-Lindahl E., Estrada S., Guan Z., Nordberg A., Langstrom B. (2009). [(11)C]PIB-amyloid binding and levels of Abeta40 and Abeta42 in postmortem brain tissue from Alzheimer patients. Neurochem. Int..

[bb0145] Tiepolt S., Hesse S., Patt M., Luthardt J., Schroeter M.L., Hoffmann K.T., Weise D., Gertz H.J., Sabri O., Barthel H. (2016). Early [18*F*]florbetaben and [11*C*]PiB PET images are a surrogate biomarker of neuronal injury in Alzheimer's disease. Eur. J. Nucl. Med. Mol. Imaging.

[bb0150] Ziegler-Graham K., Brookmeyer R., Johnson E., Arrighi H.M. (2008). Worldwide variation in the doubling time of Alzheimer's disease incidence rates. Alzheimers Dement..

